# Dynamic safety information modeling of underground cavern groups in the entire construction process

**DOI:** 10.1038/s41598-024-62928-w

**Published:** 2024-05-24

**Authors:** Hu Ankui, Wu Mengkun, Zhong Bo, Zhao Rui, Li Haizhen

**Affiliations:** 1 Key Laboratory of Fluid and Power Machinery, Xihua University, Ministry of Education, Chengdu, 610039 China; 2 Key Laboratory of Fluid Machinery and Engineering, Xihua University, Chengdu, 610039 Sichuan China; 3 School of Energy and Power Engineering, Xihua University, Chengdu, 610039 China; 4 Sichuan Fire Research Institute of Ministry of Emergency Management, Chengdu, 610036 China

**Keywords:** 4D spatial–temporal model, Dynamic safety information model, Underground cavern group, 3D visualization management, Civil engineering, Computer science

## Abstract

The construction of underground cavern groups represents a particularly challenging task in current subsurface engineering due to a multitude of variable and often unknown factors, including diverse geological conditions. This study introduces a four-dimensional spatiotemporal model and formulates a dynamic safety information model for these underground systems. Developed using C# and Python, the model integrates the finite element analysis software ABAQUS and Microsoft SQL Server database. The framework allows for real-time visual management of monitoring data, dynamic coupling of construction phases with safety metrics, and continual updates correlating with construction progress. The theoretical findings offer valuable insights for enhancing the safety and efficiency of underground cavern group construction while also supplying methods for real-time safety feedback and control throughout the construction process.

## Introduction

In numerous water conservancy and hydropower projects worldwide, including notable examples such as the Three Gorges, Xiluodu, and Jinping primary hydropower stations in China, Kolyma and Rogun in the former Soviet Union, and La Grande in Canada, significant and complex underground cavern structures are prevalent. These projects exhibit major cavern spans exceeding 20 m, with sidewall excavation heights surpassing 45 m. The construction of underground cavern complexes in such projects is marked by inherent construction uncertainties. Due to the interplay of vague geological conditions, excavation procedures, and other factors^1,2^, safety incidents within caverns during construction occur frequently. Consequently, extensive attention is directed toward the study of dynamic feedback and control of surrounding rock stability for underground cavern complexes during the construction phase.

Hoek and Brown^3^ introduced a flowchart for underground engineering design, analyzing four modes of surrounding rock instability, namely, "instability due to geological structure," "instability due to high stress," "instability due to weathering and expansion," and "instability due to groundwater pressure or seepage," tailored to different design scenarios. Brady and Brown^4^ proposed a design process incorporating multiple feedback mechanisms. Bieniawski^5^ presented a ten-step design process for underground rock excavation including modeling, data collection, problem analysis, design, and optimization, characterized by practical applicability. Pan Jiazheng^6^ proposed a comprehensive feedback analysis process for various rock engineering contexts, including steps such as "task acceptance—basic information acquisition—structural arrangement and dimension formulation—mathematical mechanical model establishment—analysis and testing—construction—operation and monitoring—verification and calibration." Zhu Bofang^7^ delineated a feedback design process during the construction phase of hydraulic structures, involving stages such as "geological exploration and material testing—technical and construction drawing design—building construction—construction data acquisition—counteranalysis—information feedback and structural design and construction program modification—project completion—operation and regulation".

Li Zhongkui et ^al.^^8^ utilized pattern search optimization techniques and nonlinear finite element methods for the nodal fissure rock model to monitor the construction process of the underground cavern group. They leveraged displacement measurements from the underground plant's monitoring system to dynamically analyze the initial ground stress for numerical calculations. Academician Cai Meifeng and other researchers^9^ summarized novel theories and techniques pertaining to nonlinear underground geotechnical structures and engineering safety monitoring. Song Zhanping et al^10,11^ initially categorized information in the tunnel engineering field based on tunnel engineering characteristics. They proposed fineness requirements for models applicable to tunnel engineering and established an initial standardized evaluation system for BIM technology in tunnel engineering, focusing on maturity and standardization.

In conventional numerical calculations for underground cavern groups, structural stability is typically assessed using predefined numerical model grids and parameters. However, underground cavern group projects are characterized by complex geological conditions, a complex array of factors influencing surrounding rock stability, incomplete and ambiguous engineering information, and dynamic changes in state and environment throughout construction progress. Most existing rock stability analysis processes do not incorporate construction progress information and measured data into the numerical calculation model. Consequently, when new geological conditions emerge, the numerical model must be reconfigured in alignment with the actual state to facilitate ongoing simulation, resulting in laborious and repetitive tasks.

**Figure 1:**
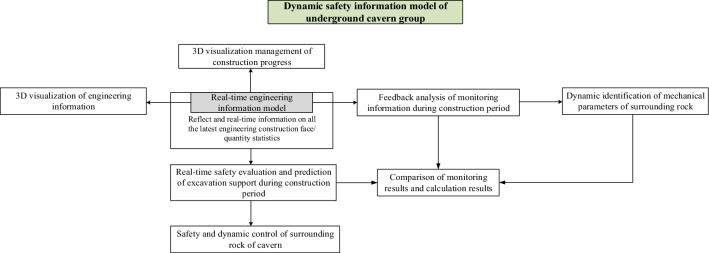
Real-time safety information model of the underground cavern group during the whole construction process-work structure.

To achieve swift, precise, and real-time representation of safety conditions within the construction environment of underground cavern groups, this study establishes a dynamic safety information model for underground cavern groups that includes the entire construction process. This model, based on a 4-dimensional spatial and temporal framework, incorporates dynamic mapping and updates of actual construction progress information, support data, and geological information. Figure 1 illustrates the structural framework of this research.

## Introduction and establishment of a four-dimensional space–time model

The primary aim of incorporating a four-dimensional spatiotemporal model into the extensive subterranean cavern group project is to augment the representation of construction progress within the temporal dimension, building upon the three-dimensional geometric model. This integration includes various facets of the stability assessment during construction, such as geological data, safety monitoring, numerical calculations, and related information. The resultant extended model establishes a dynamic mapping relationship between the construction progress and the model, enabling real-time control of the construction process and the dynamic updating and visualization of pertinent engineering data in an intuitive manner^12^. The primary objective is to simulate the entire tunnel construction process and dynamically control cavern safety.

In particular, the four-dimensional spatiotemporal model of the underground cavern group includes:

1. Three-dimensional geometric model information: Serving as the fundamental information model for the underground cavern group project, this component functions as the carrier for all other data within the information model.
2. Construction process information: This includes time-related engineering design and construction data, including construction volume, planned and actual construction progress, construction personnel, materials, machinery, costs, and more.
3. Safety information: This category includes monitoring data related to the stability of the surrounding rock of the underground cavern group, inspection data, and site survey information, among others. This paper primarily focuses on construction progress and safety monitoring data during the construction period for underground cavern group projects and discusses the methodology for creating a dynamic safety information model based on the entire construction process.

Three-dimensional geometric modeling relies on design and engineering geological information from different section directions of the extensive underground cavern group project. Utilizing three-dimensional design software, a three-dimensional solid model of the underground cavern group is constructed^13^. Subsequently, the construction area of the underground cavern group is partitioned based on construction progress information, typically categorized by the unit project under construction. The four-dimensional spatiotemporal model supplements the geometric model with dynamic construction progress-related data. Compared to the conventional three-dimensional geometric model, this model closely mirrors the actual engineering conditions. Even if construction plans change, they can be dynamically adjusted according to the unit project, thus becoming a true four-dimensional spatiotemporal model.

However, it is important to note that the construction area of the four-dimensional spatiotemporal model, as described above, introduces a time dimension and often involves an extensive number of model units, potentially reaching millions. This complexity can pose challenges for modeling and computation, sometimes exceeding the capabilities of standard computer configurations and computational resources^14^. Therefore, certain processing steps are needed for the four-dimensional spatiotemporal model.

Considering the typically large scale of underground cavern group projects, localized construction in concentrated areas may not significantly affect the stability of tunnels beyond a certain range. This study proposes the creation of a three-dimensional large-scale construction numerical model for the underground cavern group, with the following specific approach: initially, based on geological data obtained from geological exploration within the project area, a preliminary full-scale rough construction numerical model of the entire project is established, and an initial analysis of the excavation process is conducted. Subsequently, based on the structural field conditions and the evolution of the process, key safety concern areas and areas with significant group effects are identified. Furthermore, accounting for the interdependence of construction processes, several large-scale refined numerical models with dense group effects (typically at a 1.0 m unit scale) are created to meet engineering precision requirements within specific key research areas. These refined models collectively constitute the four-dimensional detailed construction information model for that particular key research area.

To develop a specific four-dimensional spatiotemporal model, several simultaneous considerations are crucial:

- Establishment of Model Scope: The excavation area serves as the central focus, with ample extension surrounding it, both horizontally and vertically (approximately 3 ~ 5 times the dimensions of the outermost chamber). The natural surface can be considered the upper boundary. This approach ensures that inaccuracies in calculation results arising from model boundaries are minimized. Furthermore, it allows for the precise simulation of group effects among the chambers^14^.
- Geological Subdivision of the Model: The geological subdivision is generally based on lithological zoning and weathering zones within the project area. For challenging geological sections such as fault fracture zones and weak interlayers within the domain of the underground cavern group project, they must be incorporated into the model. The physical and mechanical properties of rock bodies in these unfavorable geological sections differ significantly from those of the surrounding rock bodies with greater integrity.
- Excavation Construction Zoning: According to previous design information and in consideration of the progress of the underground cavern group project, the division of unit projects should be adaptable to changing program conditions. The size of the excavation units should be controlled within a 2.0 m range, and the division should ideally follow regular patterns to ensure the convergence of calculations.
- Simulation of Support Measures: The support structure for the underground cavern group primarily falls into two categories: rod structure and non-rod structure. The former includes support measures such as anchor rods, while the latter includes methods such as hanging nets, spraying, and mixing. Real-time mapping of the rod support is synchronized with the construction progress. Rod support mapping, combined with construction progress, is conducted in dynamic calculations. A preliminary three-dimensional spatial geometric model is needed for rod support mapping to facilitate the identification of the starting point for support units. Non-rod support mapping generally involves numerical simulation using equivalent rock mechanical parameters. Precision in dissected grid cells within the shallow 10.0 m range of the excavation surface is critical, with cell size controlled within the 2.0 m range. The approach for simulating equivalent support measures should align with established literature^15^.
- Simulation of Monitoring Points: Given the model's integration with monitoring information, the placement of corresponding monitoring points within the model grid must be considered. This ensures that nodes in the dynamic security information model correspond to monitoring data, offering a streamlined index for postprocessing of calculation results. For monitoring points added later in the construction phase, their location information can be linked with the nearest node in the information model^16^.

## Underground cavern group dynamic safety information model architecture

In the three-dimensional numerical calculation of underground cavern group stability, the analysis of the entire construction process typically involves three-dimensional geometric modeling, mesh division, construction step configuration, and calculations. Dynamic simulation of the construction step is achieved through "deactivation" (excavation) and "activation" (support) based on predefined cell groups. Due to the significant uncertainties in the construction process of underground cavern groups, any changes in construction progress and related information can disrupt the alignment between the predivided grid and the evolving construction conditions. Even adjusted cell sets may fail to accurately simulate the changing construction information.

Furthermore, the mechanical parameters of the surrounding rock in the numerical calculation model are typically derived from testing, introducing various uncertainty factors. The ability of these parameters to accurately reflect the mechanical behavior of the rock remains to be confirmed. Monitoring data from the construction site offers the most direct insight into the project's safety status, directly characterizing the stability of the underground cavern group. Consequently, this study will employ four-dimensional spatiotemporal modeling technology, incorporating engineering data, geological information, safety monitoring data, construction information, and more. These elements will be integrated into the dynamic feedback analysis and control of underground cavern group rock stability.

**Figure 2:**
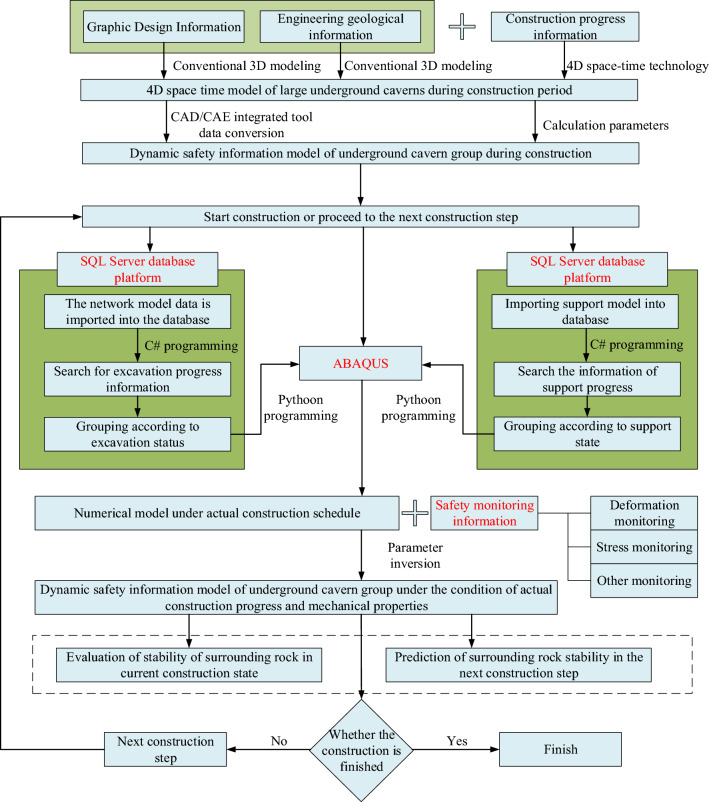
Infrastructure idea of real-time safety of underground cavern group.

A dynamic safety information model for a large underground cavern group, based on the entire construction process, is established to enable three-dimensional visualization management and dynamic assessment of cavern rock stability control. To address the limitations outlined above, the overall concept is depicted in Fig. 2.

In Fig. 2, the specific implementation steps are as follows:

- Beginning with the plan design information and engineering geological data for the extensive underground cavern group project, a conventional 3D geometric model is established. Building upon this foundation, dynamic information regarding the construction process is incorporated. Utilizing four-dimensional spatiotemporal modeling technology, a four-dimensional spatiotemporal model is created, including the entire construction process. This achieves three-dimensional dynamic visualization including the topography, geology, geomorphology, construction progress, appearance, and support measures of the extensive underground cavern group project area.
- Through CAD/CAE integration technology, a numerical simulation model containing a four-dimensional spatiotemporal component is dissected. The model's fundamental attributes are configured by defining relevant intrinsic models and calculation parameters. Notably, unlike traditional analysis methods that require numerous excavation and support units to simulate the entire construction process dynamically, the numerical simulation model based on the four-dimensional spatiotemporal model eliminates the need for this step, thus avoiding associated laborious tasks.
- In conjunction with real-time construction progress information, the potent search capabilities of the SQL database search engine are leveraged to swiftly locate the corresponding units within the calculation model for the modified areas of engineering information within the underground cavern group. A hybrid programming approach involving C#. Net and Python are employed for the secondary development of ABAQUS numerical calculation software. It automatically retrieves the search results for the units, dynamically identifies them, and modifies their rock mechanical parameters, facilitating the real-time update of the numerical calculation model for the underground cavern group.
- Recognizing that calculations based solely on parameter values within the design range may not align with the actual stability of the underground chambers, an approach employing parameter inversion techniques is adopted. This technique associates current monitoring information with calculated values for corresponding segments, establishing a mutual correction system between monitoring data and numerical simulations. Accounting for the discrete, contingent, relative, and limited nature of feedback from monitoring information in the numerical model results, it temporally and spatially aligns the numerical model's calculation results with actual monitoring results. This successfully addresses the traditional discrepancies between the two.
- A comprehensive introduction of the time factor into the dynamic numerical calculation of surrounding rock mechanical parameters for the underground cavern group is undertaken. This enables the coupling of geometric parameters, mechanical parameters, and the dynamic updates of construction information. It achieves continuous, realistic simulation in accordance with the entire construction process of the underground cavern group. This approach reveals the spatiotemporal evolution characteristics of surrounding rock mechanical parameters, employing a cyclic process that involves "prefeedback analysis results of the inversion test—instantaneous parameter analysis of dynamic inversion—prediction of surrounding rock mechanical behavior for the next excavation period".
- The analysis continues into subsequent construction phases, integrating actual construction plans and dynamic information during the construction process. The dynamic safety information of the underground cavern group is mapped until construction completion. This culminates in the dynamic feedback analysis and control of the stability of the perimeter rock of the underground cavern group, grounded in the entirety of the construction process.

## Dynamic security information model of an underground cavern group and mapping update of multisource information

### Dynamic information during construction of underground cavern complexes

The construction environment of the underground cavern cluster project involves dynamic information, including construction progress, monitoring data, and site survey findings.

- Construction Progress Information: This primarily includes updates on the excavation and support progress for each working surface within the underground cavern group construction.
- Construction Monitoring Information: Due to the complex and concealed nature of geological conditions in underground cavern group projects, coupled with the limitations of geological exploration and testing, fully revealing the project's original characteristics using conventional methods and test data is challenging. Consequently, real-time monitoring is essential during the construction of underground cavern group projects. Monitoring information plays a pivotal role in assessing the effectiveness of design and construction, preventing engineering accidents, and guiding subsequent construction^17^. To comprehensively and accurately gauge the stability and developmental trends of underground cavern projects, a diverse array of monitoring techniques is often employed. These may include deformation monitoring, strain monitoring, relaxation range monitoring, seepage monitoring, acoustic monitoring, and anchor force monitoring, among others^18^. Table 1 provides an overview of routine monitoring items for underground cavern group engineering.
- Construction Survey Information for Underground Cavern Clusters: During the excavation of the underground cavern group project, survey (observation) methods are used to gather fundamental information about the exposed areas around the cavern. This information includes details about lithology, joints, yield, faults, and the degree of slope weathering^19^. Additionally, as the cave structure is excavated, drilling is conducted to obtain data on the physical and mechanical properties of the deep geotechnical strata. This deepens our understanding of the geomechanical conditions underlying the entire cavern. In general, the primary survey information comprises inspection information. This category includes observations of deformation phenomena (such as spalling, collapse, cracks, and sliding), the condition of support structures, the state of monitoring equipment, seepage occurrences (including drainage and seepage), and the influence of blasting activities^18^. Information on New Geological Features: This pertains to newly discovered geological characteristics that may include the type, location, extent, and physical and mechanical properties of geology revealed during the construction of the underground cavern group.

**Table 1:** Monitoring items under routine conditions in the engineering monitoring of the underground cavern cluster.

Monitoring projects	Purpose of monitoring	Instrumentation
Walkaround inspection	1. The stability of the surrounding rock at the excavation face	Visual sketch, digital camera, compass, geo-radar, TSP, shallow seismometer, etc
	2. Enclosed rock structure	
	3. Deformation and stability of the surrounding rocks and supports	
	4. Calibration of perimeter rock type	
Environmental factors	Atmospheric conditions, construction conditions	Hazardous gas sensors, dust concentration sensors, noise sensors, thermometers, hygrometers, barometers, rain gauges
Surface displacement	Surface displacement of surrounding rock (convergence, vault subsidence, elevated arch bulge)	Convergence meter, total station, level, close-up photogrammetry, tunnel profiler
Internal displacement monitoring of the surrounding rock	Internal displacement of the surrounding rock (horizontal and axial)	Multipoint displacement m, borehole inclinometers, sliding micrometers, sonometers
Strain monitoring	1. Measurement of the internal strain of the surrounding rock	Strain gauge, strain brick, anchor strain gauge, anchor cable strain gauge
	2. Measurement of strain within the support (spraying and mixing)	
	3. Measurement of strain within the lining	
Stress monitoring	1. Initial stress measurement of the enclosing rock	Stress gauges (hydraulic stress gauges, differential resistance stress gauges, steel string stress gauges), reinforcement gauges
	2. Secondary stress measurement of the surrounding rock	
	3. Internal stress measurement of support and lining	
Fractures	Contact joints, cracks, structural surfaces	Crack gauge
Loading	Contact pressure between the surrounding rock and the support, anchor rod (cable) pulling force	Pressure box, pressure gauge, anchor (cable) force gauge
Demolish (using explosives)	Understanding the effects of blasting on the surrounding rock and supporting structures	Velocimeters, accelerometers
Relaxation ring observations	1. Enclosed rock blasting relaxation range measurement	Sonde, borehole multipoint displacement Gauge
	2. Measurement of the relaxation range of the secondary stress adjustment of the surrounding rock	
Seepage, groundwater	Seepage volume, seepage pressure, water table	Measuring tape, water measuring weir, seepage manometer, water level meter
Structural temperature	1. Perimeter rock temperature	General thermometer, resistance thermometer
	2. Concrete temperature	

### Updates to the mapping of dynamic security information models to multisource information

Leveraging CAD/CAE integration technology, the four-dimensional spatiotemporal model can be transformed into a grid model suitable for numerical calculations. When employing the traditional approach to simulate the construction process, manual division of cell groups within each construction step is needed. This method not only lacks the capability for automatic updates in response to construction changes but also imposes a labor-intensive workload. It becomes nearly unfeasible for complex and finely detailed numerical models.

Hence, to effectively simulate the dynamic evolution of the construction process, this study utilizes the SQL Server database platform and ABAQUS, a versatile large-scale numerical simulation tool. C#. Net programming technology is employed to identify the coordinates of excavation and support areas during construction. The units associated with construction progress are swiftly located using the robust search functionality of SQL. Through Python programming^20^, a secondary development of ABAQUS is carried out. This secondary development allows for the automatic retrieval of the identified units and their subsequent "deactivation" (excavation units) or "activation" (support units). It also enables the adjustment of corresponding mechanical parameters for the rock mass. This synchronization ensures alignment between the four-dimensional spatiotemporal model and the construction progress information. The primary technical challenges addressed include the automatic identification of construction units and their correspondence with the numerical model.

#### Secondary processing of the information model file

The correspondence between the dynamic safety information model of the underground cavern group and the construction progress information is established through the robust search functionality of the SQL server database platform. Subsequently, Python language programming is employed to perform numerical calculations on the located units, including actions such as "kill" and "activate." This necessitates the uploading of the four-dimensional spatiotemporal model into the database.

As a result, an effective mapping relationship is established between the 3D visualization-integrated engineering model and the data stored in the database. This integration allows for the seamless unification of model data and database information. It facilitates a bidirectional dynamic visualization query function, enabling users to click on the model to access data information and input data information to retrieve the specified model. This achieves the visualization management of engineering data information.

- Storage Mode of Information Model: In line with the requirements for model mapping updates, the information about the four-dimensional spatiotemporal model of underground cavern group engineering is categorized and stored in the database in the form of data tables. The classification includes node information, unit information, and material information. The breakdown is as follows: Node information includes node numbers and coordinates ($X$, $Y$, $Z$); unit information includes unit numbers, subsidiary node numbers, parent material, and unit group information, among others; and material information covers properties such as density, modulus of elasticity, Poisson's ratio, angle of internal friction, cohesion, and more.
- Input Mode of the Information Model: ABAQUS model files primarily consist of two types of files, *. cae, *. inp. Reading the numerical information model and results from the *.cae file using Python scripting can be time-consuming and cumbersome. Conversely, processing the text file *.inp is more convenient through #C program technology. This paper predominantly extracts elements of the model, including geometry nodes, coordinates, materials, and other relevant information, by parsing the *.inp file.

① Structure of the *.inp file

The file commences with a "*HEADING" tag and includes both the data section of the model (comprising details about nodes, elements, materials, element groups, node groups, initial conditions, etc.) and the parameterization section pertaining to the simulation load step. Each category of data within the.inp file is initiated by a key line, followed by a corresponding data line. Keywords must commence with the "" symbol, and comment lines are indicated by "**," as illustrated by the following example:

*Heading

……

*Part, name = PART-1

*Node

1,-2,936,855.5, 502,279.531, 1481

2,-2,936,855.75, 502,278.125, 1485.084

……

*Element, type = C3D4.

1,27,717, 25,291, 27,709, 25,292

2,27,680, 25,319, 25,318, 27,681

……

② The method of reading *.inp files

The *.inp file is processed as a text file, with the data within it identified based on the keywords present in the *.inp file. The necessary data are then extracted and stored in a structured data table in a specific format16. This process is executed using C#. Net programming as follows: Based on the storage format of the data table, create a "Node" class and a "Unit" class. The "Node" class includes node numbers and coordinate information (x, y, z), while the "Unit" class includes unit numbers and subsidiary node numbers. Proceed to read the needed information from the *.inp file according to keywords such as NODE, ELEMENT, and MATERIAL. For node, unit, and material information, it is necessary to identify the key fields and subsequently upload them to the database.

#### Dynamic identification of excavation information

The primary challenge in conducting a stability analysis of the surrounding rock, based on the dynamic safety information model of the underground cavern group, lies in the ability of the numerical model used for analysis to faithfully and objectively simulate the actual engineering conditions with the requisite accuracy to meet engineering standards. In this paper, an enhancement is proposed by introducing the concept of "set" mapping. This improved mapping update algorithm facilitates the visualization, querying, and management of excavation volume and excavation progress.

(a) Mapping Update Concept: Mapping, in this context, pertains to establishing a correspondence between two sets. When applied to real-time tunnel simulation and analysis, it can be expressed as follows in Eq:

1 $$ Y{ = }f\left( X \right) $$

where X is a set of construction site information; Y is a set of numerical simulation model information; and *f* is the mapping function.

(b) Mapping of excavation progress: Considering the nature of the underground cavern group project, where excavation progresses layer by layer from top to bottom, each layer of excavation represents a distinct unit project. Given that the size of the excavation unit aligns with mapping accuracy requirements, the completed excavation area is linked to the corresponding excavation unit for each phased excavation step through a "collection" process. Subsequently, the units are "deactivated" in accordance with the sequencing of phased excavation to facilitate the simulation analysis of construction progress. The mapping of excavation progress is contingent on the status of each unit project, primarily falling into three categories: excavated, under excavation, and unexcavated. The specific implementation methods are as follows:

① Mapping of excavated completed units

Identify unit projects that have completed excavation, which are defined as those where the current date specified is greater than the actual end date of excavation for the unit project. Subsequently, search for model cells associated with these completed unit projects from among all excavation cells within the numerical calculation model. This search is conducted based on the location information, including the elevation range and pile number, for each completed unit project in succession.

The modeling unit numbers corresponding to each unit project upon the completion of excavation are stored in the "excavated modeling unit collection finish" ${E}_{finish-s}$.

② Mapping of unit work being excavated

Identify unit projects that are currently under excavation, which are defined as those where the specified current date is greater than the actual start date of excavation for the unit project and less than the actual end date of excavation for the unit project. Subsequently, search for model units associated with each of these ongoing unit projects, one project at a time.

Based on the excavated footage or work volume of the unit project (retrieved from the actual progress information table for the unit project), select the model unit that corresponds to the excavated portion from among all the model units within the unit project. Store the number of the model unit in the "set ${E}_{finish-s}$ of excavated model units".

③ The excavated cells are mapped to the numerical model

The units within the numerical model are matched to the units within the numerical model based on the unit numbers found in the "excavated model unit set ${E}_{finish-s}$." Subsequently, the life-and-death unit method is employed to deactivate the units that have been excavated.

#### Recognition of support progress information

Considering the actual engineering aspects of the underground cavern group and the characteristics of the support structure, the mapping of support progress includes both rod support measures such as anchor rods and anchor cables, as well as nonrod support measures such as hanging nets and sprayed concrete.

**Figure 3:**
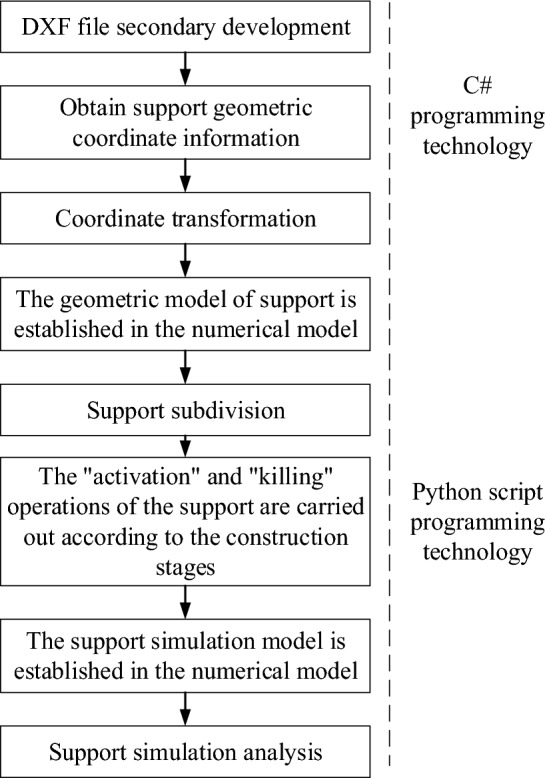
Update flow of the real-time map of support.

Mapping Rod Support: Rod support measures are mapped through solid element modeling. The mapping method is depicted in Fig. 3.

The concept for real-time mapping of the support schedule is as follows: In accordance with the actual phased support progress of the tunnel project, a DXF text file serves as the medium for mapping the support "set." Each layer's support information within the file is treated as an "element" within the support "set." After recognizing the support geometric information, performing three-dimensional coordinate conversions, support modeling, support simulation, and related operations, the phased support information contained in the DXF file is then mapped to the numerical simulation model. This enables real-time simulation and analysis of the construction support progress state.

The key and challenging aspects of updating support mapping can be primarily categorized into two points:

Recognition of Support Geometric Model Information: Proficiency in understanding the significance and the relationship between the code snippet and group code within the DXF is essential. Utilizing C# programming technology, a secondary development of the DXF file is carried out to identify the geometric information of the support model on each layer.

Real-time Establishment of Support Simulation Model: Leveraging Python script programming technology, the secondary development of ABAQUSQ numerical software is initiated. Employing a bottom-up modeling approach, the support simulation model is established. Under the support's stage load step, the process of deactivation and activation of support is initiated, ultimately leading to the dynamic updating of support progress.

Nonrod support mapping, on the other hand, involves the utilization of equivalent mechanical parameters for the surrounding rock of the underground cavern group. The determination of the equivalent area is primarily based on prior grouping based on experience^15,21^.

#### Dynamic coupling of monitoring information

To incorporate the actual monitoring data of the project into the dynamic safety information model of the underground cavern group and enable visualization, querying, and management of safety monitoring data, a method that combines uniform design and neural network intelligent optimization algorithms is employed for the dynamic identification of surrounding rock mechanical parameters^22^. This approach involves dynamically adjusting the originally designed physical and mechanical parameters, establishing a mutual correction system between monitoring information and numerical simulations, and comprehensively introducing the time factor into the dynamic numerical simulation and identification of the surrounding rock mechanical parameters of the underground cavern group. This process enables the coupling of geometric and mechanical parameters and the dynamic updating of construction information.

**Figure 4:**
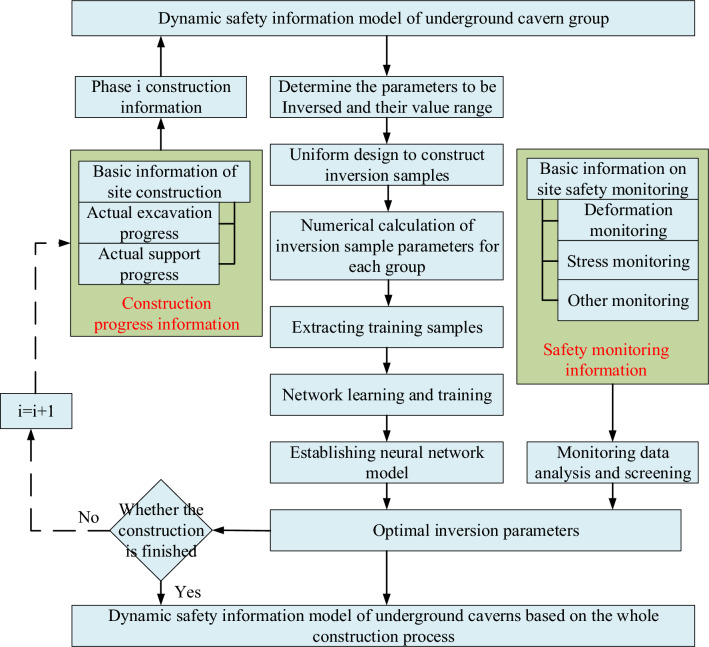
Real-time flow of the real-time safety information model of the underground cavern group rock mechanical parameters.

The coupling between geometric parameters, mechanical parameters, and the dynamic updating of construction information not only unveils the evolutionary characteristics of the spatial and temporal properties of the surrounding rock mechanical parameters but also aligns and correlates the numerical model's calculation results with the actual monitoring results in both space and time. The specific process is illustrated in Fig. 4.

The dynamic inversion of the mechanical parameters of the surrounding rock of the underground cavern group follows these steps:

*STEP 1* Begin by establishing the initial numerical simulation model, which is based on the inversion of the initial ground stress field ^23^and incorporates information such as the layout of the cavern group, structural dimensions, and topographic and geological data of the plant area.

*STEP 2* Dynamically modify the 3D numerical computation model based on actual excavation progress, support progress, newly exposed geological data, and other site information for the current construction period (construction period i) of the underground cavern group. This results in a real-time numerical simulation model for period i that aligns with the construction site conditions.

*STEP 3* Conduct the identification of mechanical parameters for the rock body in Phase i and define the optimization inversion objective function.

*STEP 4* Determine the tentative surrounding rock mechanical parameters using the principle of parameter sensitivity^24^. Design an experimental program based on the suggested value range of these parameters, employing the uniform design method. Obtain training and validation samples for the artificial neural network (ANN) algorithm through numerical computations with ABAQUS.

*STEP 5* Employ the ANN optimization algorithm to learn from the training samples established in STEP 4. This establishes the nonlinear mapping relationship between input values and output values.

*STEP 6* After outlier processing of monitoring displacement^25^, utilize the neural network's machine learning function with the actual monitoring displacement information as input to obtain optimal rock mechanical parameters. This step signifies the completion of identifying the mechanical parameters of the surrounding rock in the current Phase i.

*STEP 7* If the excavation construction of the underground cavern group is not yet completed, return to STEP 2 to initiate the inversion of the mechanical parameters of the surrounding rock for the next period. This process is continued until the construction of the cavern group is finished, and the inversion process is completed.

At this stage, the dynamic safety information model for a large underground cavern group includes three-dimensional visualization of engineering data, dynamic mapping of construction progress information, processing of safety monitoring data, dynamic identification of surrounding rock mechanical parameters, and assessment and prediction of the stability of excavation and support for the surrounding rock during the construction period based on numerical simulations. This model serves the following main purposes:

Stability Evaluation: This assesses the stability of each part of the underground cavern group in its current state. Since monitoring information reflects only local areas, the dynamic safety information model can provide a better representation of the dynamic safety and rock stability of different engineering areas within the cavern group.

Prediction and Evaluation: It predicts and evaluates the stability of the next construction step. In cases of local instability, numerical calculations can be used to optimize the proposed support schemes, addressing practical challenges in the project.

## Engineering application

This research utilizes the Huangdeng Hydropower Station project as a representative example to demonstrate the methodology for establishing a dynamic safety information model based on the entire construction process. The development of this model leverages large-scale general finite element analysis software, ABAQUS, and Microsoft SQL Server database software. Programming languages such as C# and Python are used to create the dynamic safety information model for the underground cavern group, enabling real-time feedback analysis and control of surrounding rock stability during the construction period.

The primary functions of this model include the dynamic updating of a four-dimensional spatiotemporal information model for the underground cavern group, three-dimensional visualization of construction progress, analysis of monitoring data during construction, dynamic identification of mechanical parameters for the surrounding rock, and real-time assessment and prediction of the safety of various components, particularly the surrounding rock.

Huangdeng Hydropower Station is a significant water conservancy and hydropower project primarily focused on power generation. It offers comprehensive benefits such as flood control, irrigation, water supply, soil and water conservation, and tourism. The hydropower station is situated in Lanping County, Yunnan Province, China, in the upper reaches of the Lancang River. The diversion power generation system is located on the left bank of the hub, with a complex layout of underground caverns that includes the main and auxiliary workshops, main transformer room, tailwater overhaul gate room, bus tunnel, pressure pipeline, tailrace branch tunnel, and tailwater tunnel. The geography of the pivotal area features a narrow "V"-shaped river valley, steep slopes with natural angles greater than 45 degrees, and irregular bank slope topography characterized by developed gullies and intersecting gully beams.

### Dynamic updating of a four-dimensional spatial–temporal information model for underground caverns

**Figure 5:**
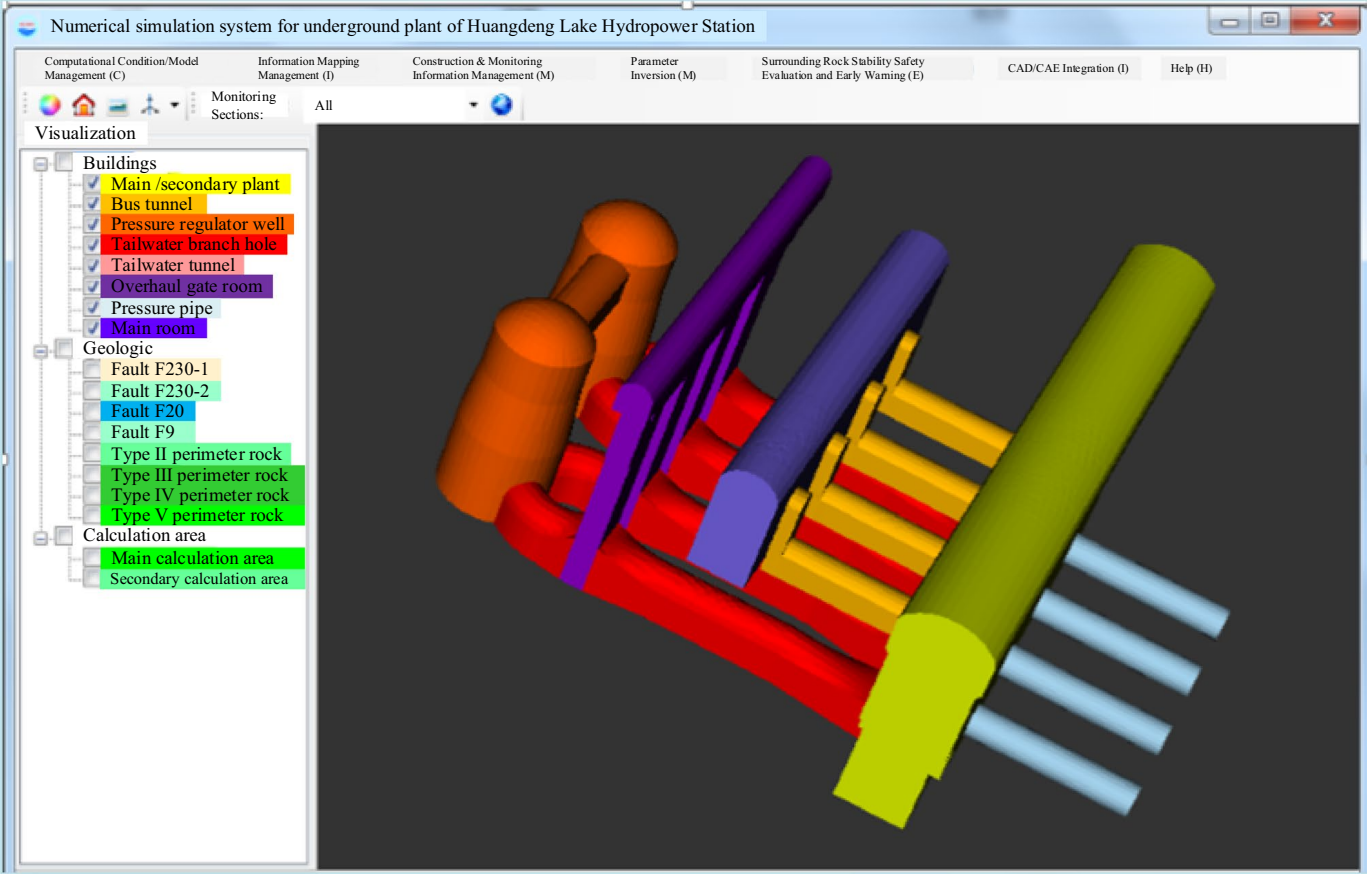
3D visualization model of the HuangDeng hydropower station underground cavern group.

Within the real-time numerical simulation system for tunnel engineering, the real-time mapping of tunnel conditions is a pivotal element in the methodology examined in this research. This mapping process is essential for conveying up-to-the-min construction status information and includes three primary aspects: three-dimensional visualization of engineering data, dynamic mapping of newly exposed geological features, and real-time updates. Figure 5 illustrates the three-dimensional visualization model of the underground cavern group at Huangdeng Hydropower Station, providing a comprehensive view of the project.

**Figure 6:**
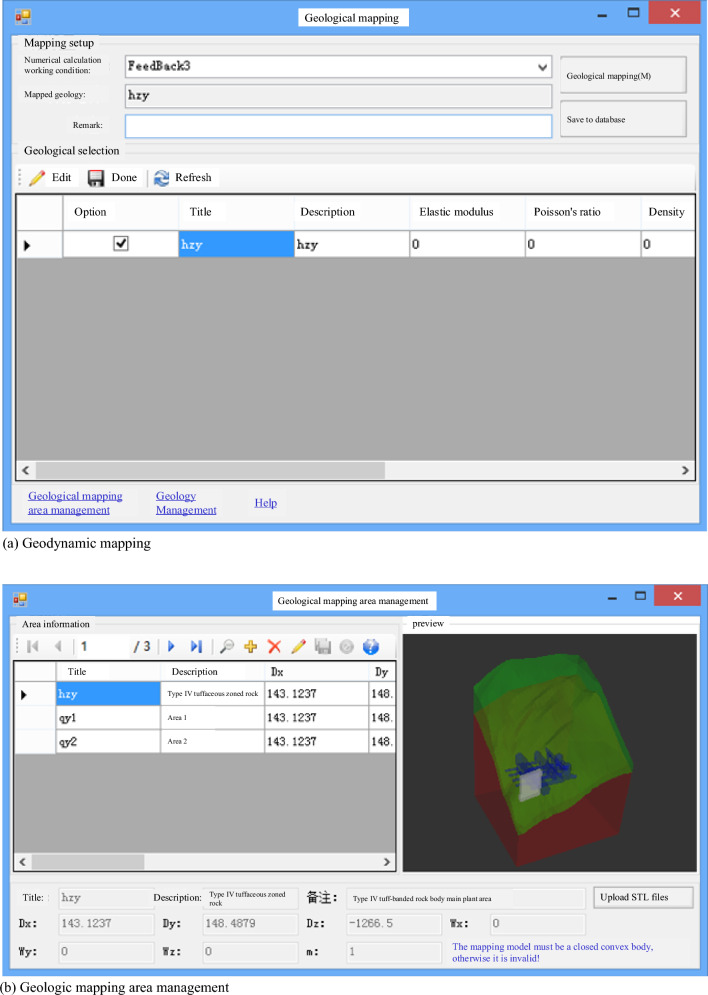
Updated dynamic mapping of the newly exposed geology of the underground cavern group of the Huangdeng hydropower station.

However, it is crucial to address situations where unforeseen or newly discovered geological information emerges during excavation. In such cases, this information must be promptly and dynamically incorporated into the numerical simulation model. The operational interface for this updating process is depicted in Fig. 6.

### 3D visualization management of construction schedule

**Figure 7:**
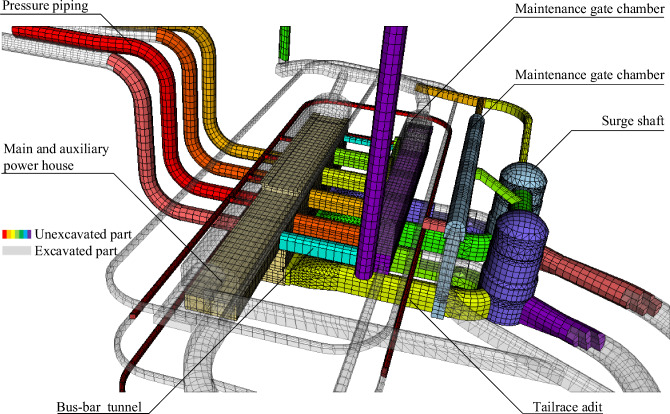
3D visualization of construction progress.

Construction progress is primarily depicted in the excavation and support phases within the various caverns of the numerical model, as illustrated in Fig. 7. In this representation, the white portion represents sections of the cavern that have undergone excavation, while the colored segments signify areas that remain unexcavated. The automatic mapping of progress information, which involves dividing the excavation into unit engineering segments, is accomplished through backend calculations.

For real-time updates related to support and other factors, a combination of C# and Python scripting technologies is employed to facilitate the mapping of support schedules. This mapping includes both rod support measures, such as anchor rods and anchor cables, and nonrod support measures, including hanging nets and spraying concrete. Table 2 in the compiled database outlines the data format for relevant construction progress information.

**Table 2:** Data format of construction progress information of tunnel engineering.

Field name	Data type	Field name	Data type
Unit engineering number	nvarchar	Unit engineering name	nvarchar
Underground cavern engineering	nvarchar	Ownership segment	nvarchar
Top elevation	float	Bottom elevation	float
Initial dam transverse	float	Terminating dam transverse	float
Construction organization	nvarchar	Planned duration	float
Design length	float	Actual excavation advance	float
Total planned excavation	float	Actual total excavation	float
Planned excavation start date	datetime	Actual excavation start date	date
Planned excavation end date	datetime	Actual end date of excavation	date
Planned support start date	datetime	Actual support start date	date
Completion date of planned support	datetime	Actual support completion date	date
Planned support type	nvarchar	Actual support type	nvarchar
Acceptance date	date		

**Figure 8:**
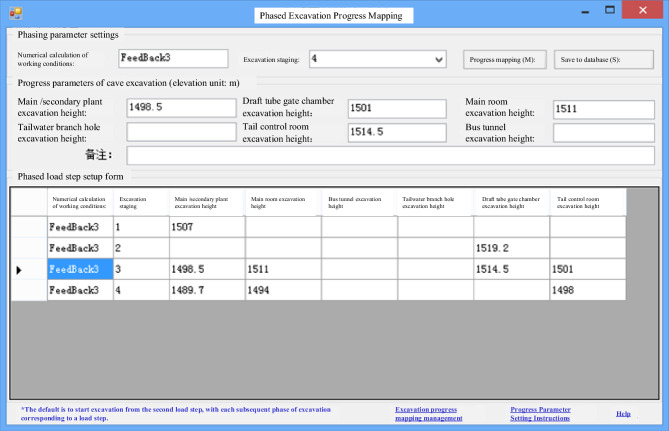
Progress mapping of phased excavation of underground cavern cluster.

**Figure 9:**
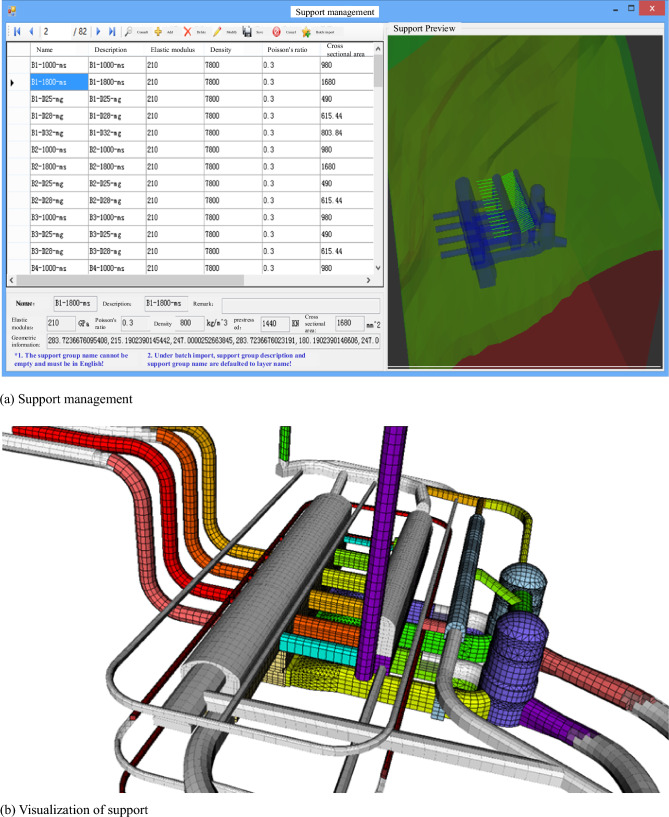
Dynamic mapping management of underground cavern group support in the Huangdeng hydropower station.

The dynamic mapping of the excavation progress and support management is presented in Figs. 8 and 9, respectively. In Fig. 9(b), the silver-white area denotes complete support, while the colored regions indicate areas awaiting support.

### Feedback analysis of monitoring information during construction

Upon achieving the dynamic update of the four-dimensional spatiotemporal model for the underground cavern group, reflecting the dynamic safety information of the information model entails integrating monitoring information into the model for the scientific management of construction monitoring data.

Despite significant advancements in theoretical rock engineering analysis, the practical experience of engineers and technicians, as well as on-site intuitive judgment and monitoring capabilities, remain vital for ensuring the safety of rock engineering^26^. In many excavation projects, the ultimate decisions often rely on experience and wise judgment for intuitive evaluation.

**Figure 10:**
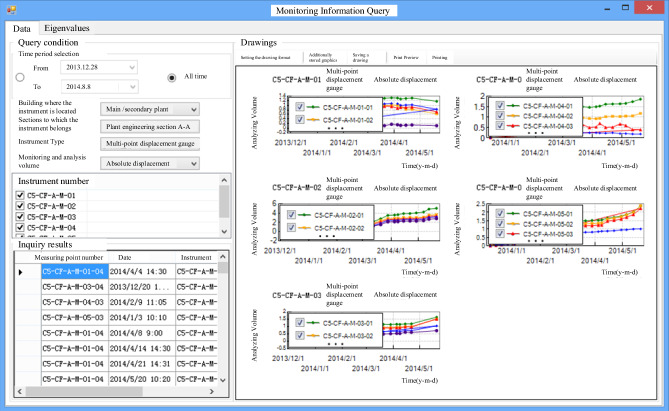
Query of monitoring information.

**Figure 11:**
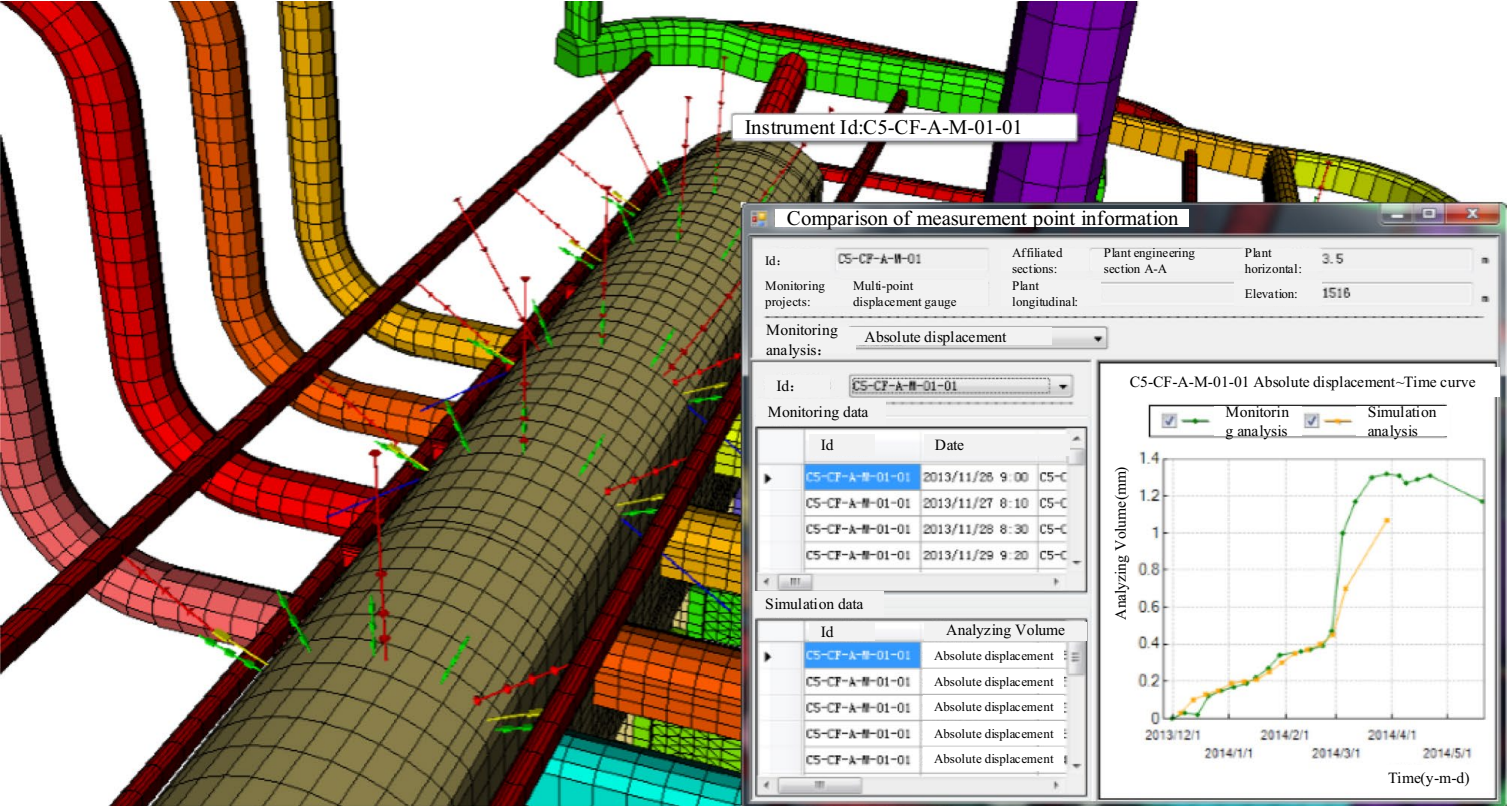
Visualization of monitoring equipment.

**Figure 12:**
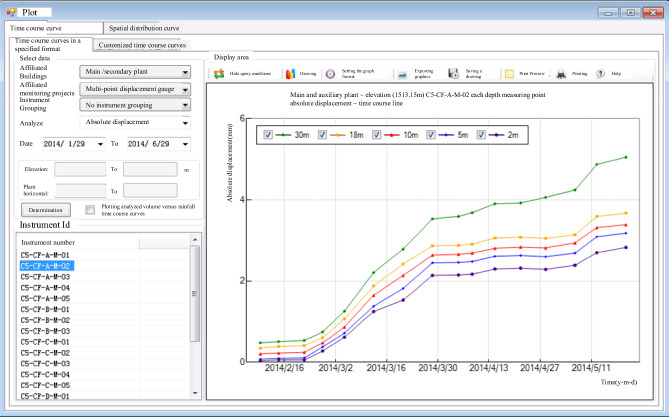
Monitoring data of time curve analysis.

As depicted in Figs. 10 and 11, the process involves querying data from each monitoring instrument and dynamically managing these instruments. This information is then used to generate time-course curves of measured data and perform related eigenvalue analyses, as shown in Fig. 12. This approach allows for the rapid evaluation of the stability of the surrounding rock in the underground cavern group, thereby ensuring the safety of the construction period and the normal operation of the project.

### Dynamic identification of the mechanical parameters of the surrounding rock

**Figure 13:**
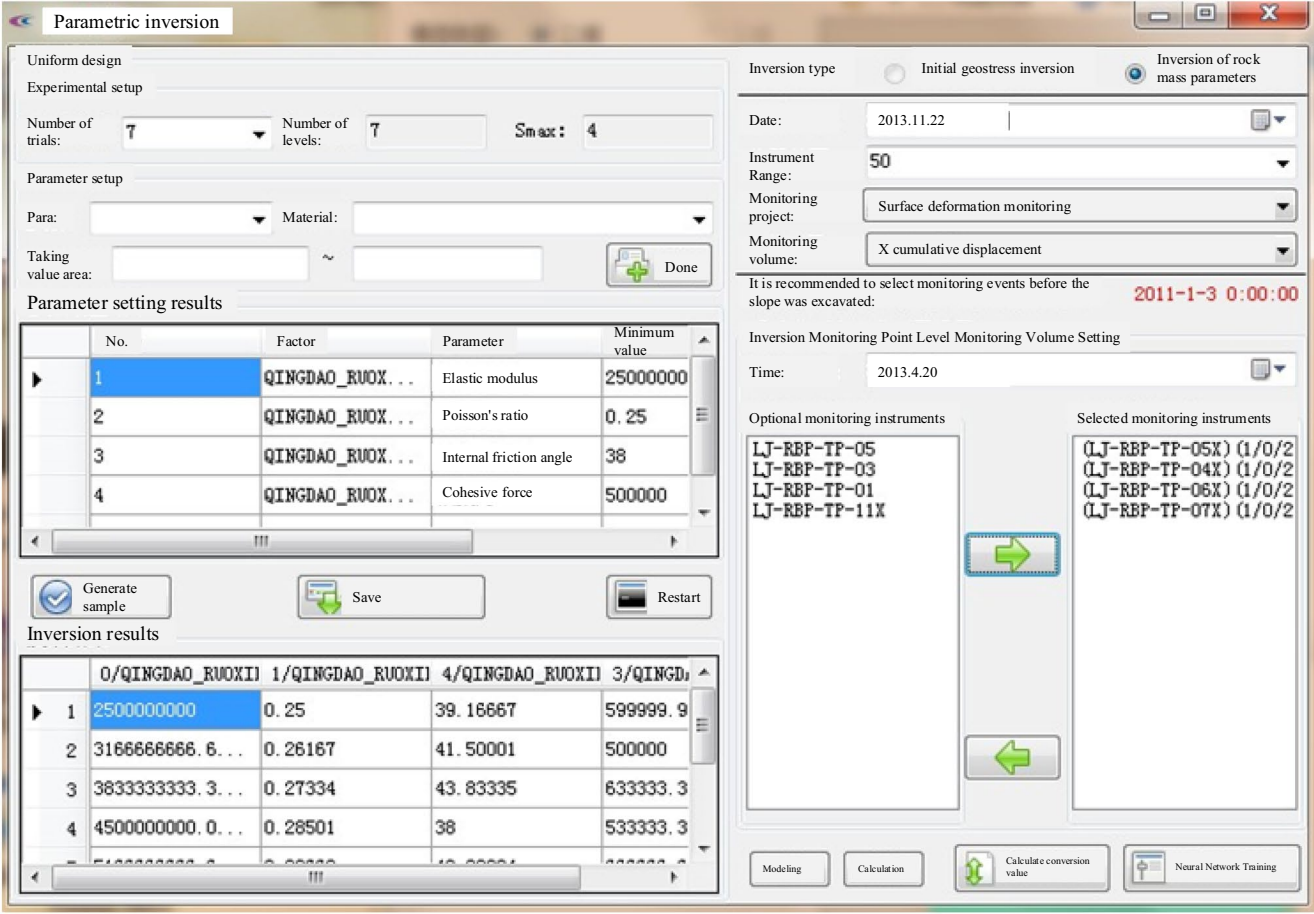
Dynamic recognition interface of the mechanical parameters of the perimeter rock.

As examined in section "Dynamic coupling of monitoring information", the focus is on dynamic identification based on the mechanical properties of the surrounding rock during the construction phase of the underground cavern group. Initially, the ranges for the parameters to be inverted are established. Subsequently, inversion samples are generated via orthogonal or uniform design testing methods. The computed values for the monitoring points of each sample set are obtained through the cyclic automatic numerical calculation function designed for inversion samples. Following this, these values serve as input for an ANN optimization model, which is trained until it meets the specified accuracy criteria. Once training is complete, the actual monitoring values are input into the ANN algorithm for optimization, yielding the inversion results. The interface for the dynamic identification of mechanical parameters is depicted in Fig. 13.

### Real-time evaluation and prediction of the surrounding rock safety of underground caverns

**Figure 14:**
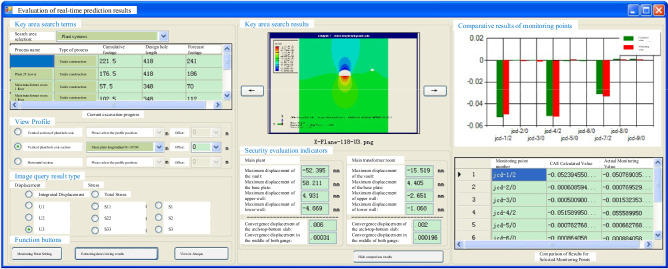
Real-time evaluation of underground cavern group rock.

The dynamic safety information model of the underground cavern group based on the entire construction process serves the purpose of conducting real-time evaluation and forecasting of the surrounding rock's safety. The results obtained from numerical calculations are processed, preprocessed, and incorporated into the database through compilation. This data compilation interface for excavation performs functions such as compilation, preprocessing, and inputting the results obtained from finite element analysis software into the database. It also extracts numerical calculation results related to specified monitoring points and monitoring parameters. It facilitates the visual comparison, querying, and generation of curves representing monitoring data. Additionally, it enables intelligent analysis of the reasons for comparison results, providing valuable support for construction management personnel in their decision-making processes. The corresponding interface design is illustrated in Fig. 14.

**Figure 15:**
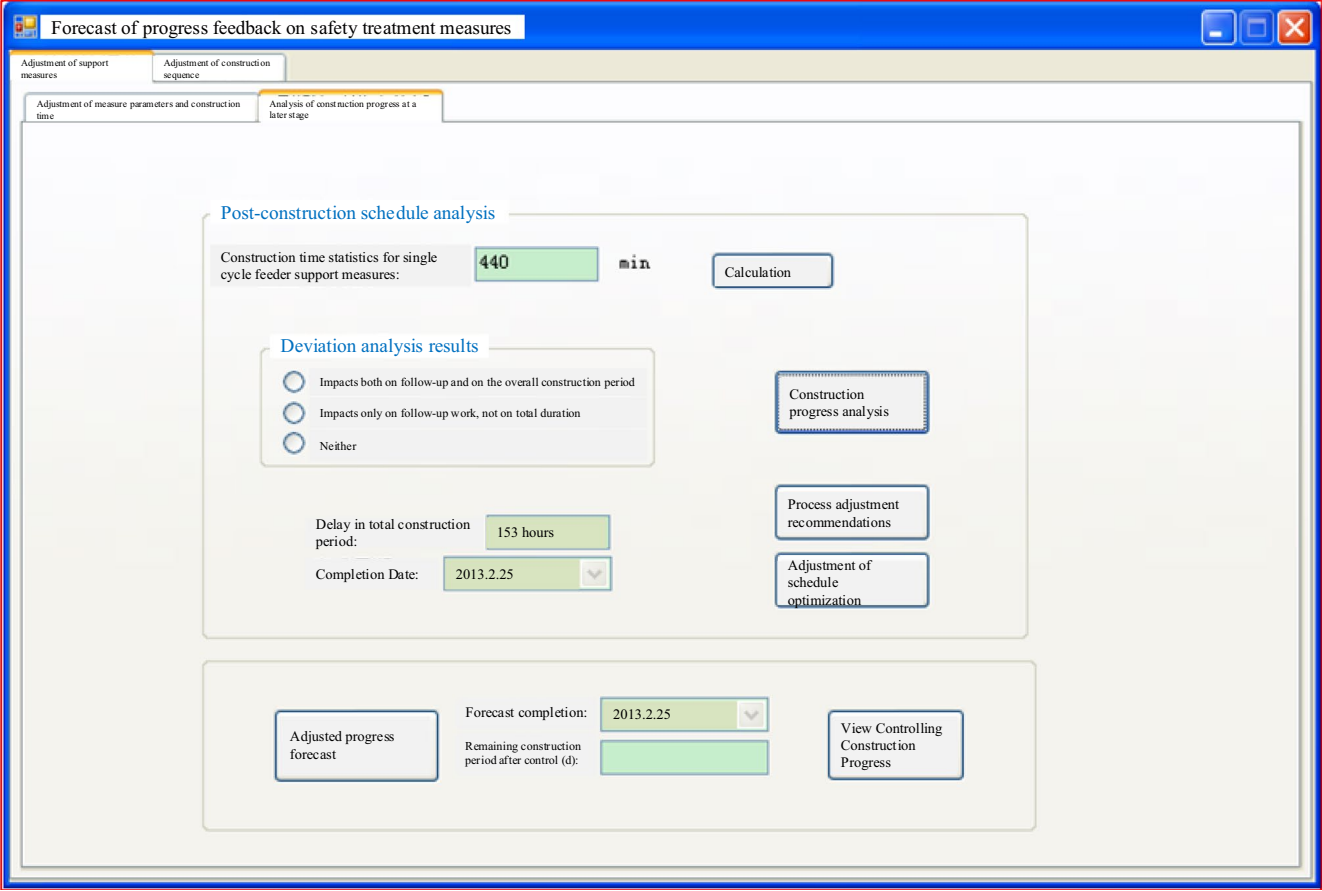
Support program feedback construction schedule prediction interface.

Comprehensive dynamic numerical calculation results, safety assessment indicators, and other relevant data are combined with the project's engineering expertise to assess the ongoing safety of the structure. If it is determined that the surrounding rock does not meet the safety and stability requirements, appropriate remedial measures are identified. These measures are then integrated with the chosen support strategies after considering construction progress prediction and correction, as depicted in Fig. 15. This approach ensures real-time safety assessment and intervention as needed to maintain the project's safety standards.

## Conclusion and perspective

### Conclusion

This paper establishes a dynamic safety information model for underground cavern groups, including the entire construction process. It integrates visualization technology, monitoring and analysis theory, parameter inversion theory, numerical simulation technology, and computer technology to achieve the visual management of monitoring information related to underground cavern groups. This model dynamically couples the construction progress with the visualization of the cavern safety status and continuously updates construction information.

Upon application to the Huangdeng Hydropower Station underground cavern group project, the following key findings emerge:

- Integrating a four-dimensional spatiotemporal model into the dynamic feedback analysis and control of surrounding rock stability throughout the underground cavern group construction process proves highly effective. This approach provides a safety information model that adapts to construction progress, facilitating visual engineering information management. It enables dynamic simulation of the entire excavation and support process, thereby enhancing calculation and analysis efficiency. Moreover, it offers practical insights and analytical methods for the entire construction process of underground cavern groups.
- Embracing the concept of three-dimensional dynamic visualization, this model achieves comprehensive and full-scale representation of the geological landscape from all angles. It dynamically displays construction progress, monitoring instruments, project volume, and safety visualization. By incorporating real-time mapping of construction progress, geological data, and support information during construction, a real-time safety feedback analysis and control approach for cave chambers based on numerical simulation technology is proposed.
- The temporal aspect is complexly integrated into the dynamic numerical calculation of mechanical parameters for the surrounding rock of underground cavern groups. This allows for the coupling of geometric and mechanical parameters with the dynamic update of construction information. The continuous and all-including simulation of the entire underground cavern group construction process unveils the temporal and spatial characteristics of the mechanical parameters of the surrounding rock.

### Perspective

In the development of the dynamic safety information model for underground cavern groups, while we do consider dynamically mapping and updating excavation progress, support information, and newly exposed geological features, there are still significant amounts of unforeseeable factors, such as collapses and cracks. It is crucial to further investigate methods for accurately incorporating these unpredictable elements into the numerical model.

## Data Availability

The data used to support the findings of this study are included in the article.
